# Innate immune memory in cardiometabolic disease

**DOI:** 10.1093/cvr/cvad030

**Published:** 2023-02-16

**Authors:** Harsh Bahrar, Siroon Bekkering, Rinke Stienstra, Mihai G Netea, Niels P Riksen

**Affiliations:** Radboud University Medical Center, Department of Internal Medicine and Radboud Institute for Molecular Life Sciences, 463, Geert Grooteplein Zuid 8, 6525 GA Nijmegen, The Netherlands; Radboud University Medical Center, Department of Internal Medicine and Radboud Institute for Molecular Life Sciences, 463, Geert Grooteplein Zuid 8, 6525 GA Nijmegen, The Netherlands; Radboud University Medical Center, Department of Internal Medicine and Radboud Institute for Molecular Life Sciences, 463, Geert Grooteplein Zuid 8, 6525 GA Nijmegen, The Netherlands; Nutrition, Metabolism and Genomics Group, Division of Human Nutrition and Health, Wageningen University, Wageningen, The Netherlands; Radboud University Medical Center, Department of Internal Medicine and Radboud Institute for Molecular Life Sciences, 463, Geert Grooteplein Zuid 8, 6525 GA Nijmegen, The Netherlands; Department of Immunology and Metabolism, Life and Medical Sciences Institute, University of Bonn, Bonn, Germany; Radboud University Medical Center, Department of Internal Medicine and Radboud Institute for Molecular Life Sciences, 463, Geert Grooteplein Zuid 8, 6525 GA Nijmegen, The Netherlands

**Keywords:** Atherosclerosis, Obesity, Inflammation, Monocytes, Trained immunity

## Abstract

Low-grade systemic inflammation is a key pathophysiological component of atherosclerotic cardiovascular disease (CVD), and long-term activation of myeloid cells is thought to be crucial for these effects. Obesity and associated metabolic complications including hyperglycaemia and dyslipoproteinaemia can induce long-lasting inflammatory reprogramming of the innate immune cells and their bone marrow progenitors, which in turn contributes to atherosclerosis. In this review, we discuss the mechanisms through which innate immune cells undergo long-term changes in their functional, epigenetic, and metabolic characteristics upon even short-term exposure to endogenous ligands, a process also termed ‘*trained immunity*’. Inappropriate induction of trained immunity leads to the development of long-lasting hyperinflammatory and proatherogenic changes in monocytes and macrophages, an important factor in the development of atherosclerosis and CVDs. Knowledge of the specific immune cells and the distinct intracellular molecular pathways involved in the induction of trained immunity will reveal novel pharmacological targets that could be used to prevent or treat CVDs in the future.

**This article is part of the Spotlight Issue on Obesity, Metabolism, and Diabetes.**

## 1. Introduction

**Figure 1:**
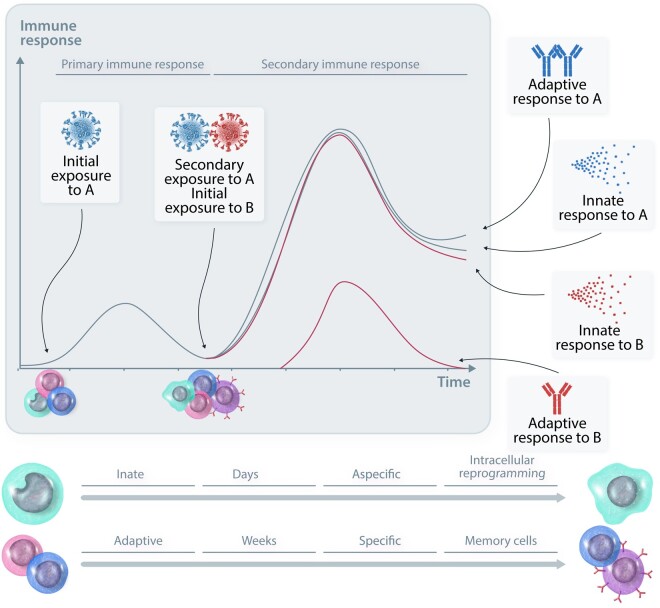
Differences and similarities in innate and adaptive memory. Upon a first initial exposure to stimulus A, cells of the innate and adaptive immune system are both activated. Within days, innate immune cells can develop a memory via intracellular reprogramming. Upon secondary stimulation with either stimulus A or an unrelated stimulus B (heterologous), an increased immune response is observed. Adaptive immune memory takes longer to establish (weeks), but is very specific and lasts long through the formation of memory cells. However, only restimulation with stimulus A leads to an enhanced antibody response; introducing a new stimulus (B) leads to a new ‘primary’ immune response.

The innate and adaptive immunity components of host defence act in close interaction to defend the host against infections, perform immune surveillance, and mediate tissue repair after injury.^1^ For a long time, the main differences considered to distinguish between these two arms of immunity were the antigen-dependent specificity and the long-term memory to specific pathogens, both properties considered to characterize exclusively the adaptive immunity.^2^ Recent evidence suggests, however, that not only the adaptive immune response, but also innate immune cells can display memory of a previous infection or injury, with differences persisting at the level of specificity and durability of these traits (*Figure 1*).

Innate immune memory, also termed ‘*trained immunity’*, is characterized by long-term functional reprogramming of innate immune cells that results in more effective antimicrobial functions upon encountering a secondary pathogen: increased cytokine production and reactive oxygen species, and improved phagocytosis and killing of the pathogen.^3,4^ The last decade has been the witness of major progresses in understanding the mechanisms mediating trained immunity. At a molecular level, trained immunity is mediated by long-term epigenetic reprogramming through modulation of chromatin accessibility and DNA methylation, which licenses a more effective gene transcription upon restimulation.^5,6^ Rewiring of cellular metabolism, with increased glycolysis, oxidative phosphorylation (OXPHOS), and activation of the cholesterol synthesis pathway, as well as repurposing of glutaminolysis and Krebs cycle with the accumulation of metabolites that modulate epigenetic enzymes (such as succinate and fumarate) are also important mechanisms conveying trained immunity.^7–9^ At the cellular level, trained immunity can be induced in both myeloid and lymphoid innate immune cell populations. All major myeloid cell populations such as monocytes, macrophages, and neutrophils have been demonstrated to undergo changes that result in an increased function after exposure to infections, vaccinations, or injury.^10–12^ In addition, NK-cell and innate lymphoid cells can also mount memory properties in an antigen-independent manner.^13,14^ Trained immunity is triggered both at the level of bone marrow precursors (central trained immunity; see accompanying review by Mitroulis *et al.*^15^) and at the level of the effector cells with a long lifetime in tissues such as macrophages (peripheral trained immunity).^10,16,17^ Importantly, a trained immune phenotype has also been described in non-immune cells such as endothelial or epithelial cells.^18,19^

While trained immunity can induce heterologous protection against infections for several years after administration of live attenuated vaccines,^20,21^ recent studies have demonstrated that inappropriate activation of these mechanisms in immune cells can also lead to autoinflammatory processes and disease.^22^ In this respect, trained immunity can also be induced by certain endogenous stimuli such as oxidized lipids, metabolites, or uric acid crystals. Subsequent activation of similar epigenetic and molecular mechanisms as those that underlie the training of immune cells by micro-organisms can lead to inappropriate inflammation that contributes to the pathophysiology of many inflammatory and auto-immune diseases, among which are gout, systemic lupus erythematosus, rheumatic and neurodegenerative diseases, and atherosclerosis.^22^ In this review, we will describe the impact of trained immunity processes involved in the pathogenesis of atherosclerosis and cardiovascular diseases (CVDs), in particular in the context of obesity, metabolic syndrome, and diabetes. We will focus on these processes on the tissue/peripheral level, whereas the accompanying paper by Mitroulis *et al*. describes the role of central trained immunity at the level of bone marrow progenitors in more detail.^15^

We will first summarize the intracellular molecular mechanisms that are essential for the development of trained immunity. Subsequently, we report the role of inflammation and innate immunity in obesity, diabetes, and atherosclerosis, and finally, describe in detail which stimuli and intracellular pathways can drive trained immunity in the context of these disorders.

## 2. Mechanisms of trained innate immunity

To date, two major molecular mechanisms underlying the induction of innate immune memory have been described: rewiring of key metabolic pathways and long-term epigenetic changes. The exact pathways and affected genes differ per stimulus and there are differences between exogenous and endogenous stimuli that induce trained immunity. Other mechanisms, such as post-translational (m6A) RNA or protein modifications may also be involved in the induction of trained immunity, but further research is needed to assess the importance of these additional pathways.^23^ Importantly, most knowledge about these mechanisms has been obtained from *in vitro* experiments in which isolated primary human monocytes were exposed to a training stimulus [e.g. microbial stimuli such as *Candida albicans* and Bacille Calmette–Guerin (BCG), or metabolic stimuli such as oxidized low-density lipoprotein (oxLDL) or uric acid crystals] for 24 h, and restimulated 6 days later with a heterologous stimulus [e.g. lipopolysaccharide (LPS)] to assess cytokine production capacity or intracellular metabolic or epigenetic changes.^24^

### 2.1 Immunometabolism as the guard of (innate) immune cell function

Over the last decade, technological advances have helped boost research into metabolic processes that influence immune response and which—as a result—highly increased our knowledge of innate immune cell function and how immune cells respond to external (danger) stimuli. Six major metabolic pathways are important for innate immune cell activation: glycolysis, the tricarboxylic acid (TCA) cycle that fuels OXPHOS, the pentose phosphate pathway (PPP), fatty acid oxidation, fatty acid synthesis (FAS), and amino acid metabolism.^25^ All these pathways are key to activating immune cells, producing energy, and biosynthetic building blocks, and the production of cytokines for a quick immunological response upon stimulation. Different stimuli induce different pathways, resulting in either pro- or anti-inflammatory responses. The pathways are also connected, since intermediate products of one pathway feed into the other. In addition, the metabolites of several pathways are also necessary for roles outside metabolism, e.g. to regulate epigenetic enzymes.^5^

### 2.2 Metabolic rewiring in trained innate immune cells

**Figure 2:**
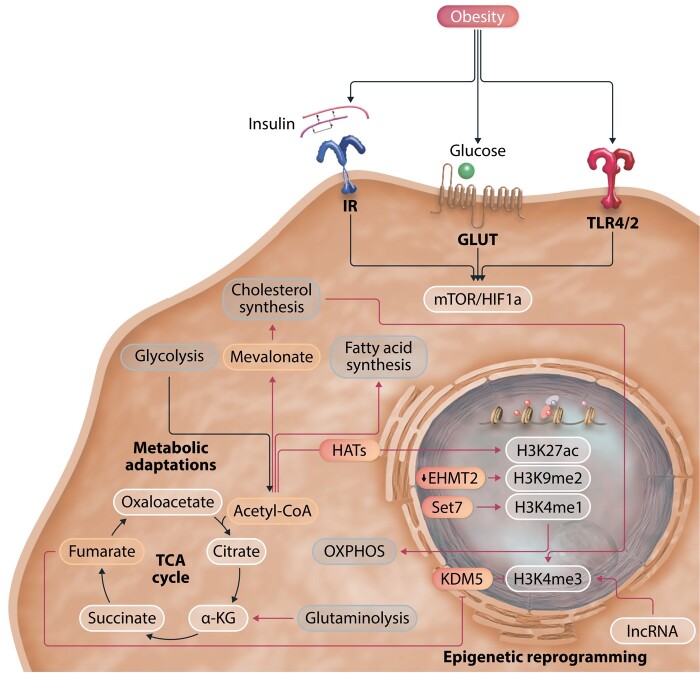
Mechanisms of trained immunity. Upon initial stimulation, innate immune cells undergo activation and subsequent metabolic adaptations, involving up-regulation of glycolysis, glutaminolysis, OXPHOS, the TCA cycle, FAS, or Cholesterol synthesis, depending on the stimulus. Metabolic rewiring is intertwined with epigenetic programming, as metabolites of several pathways influence epigenetic marks or erasers, for example, via Fumarate and KDM5. Known receptors of innate immune activation in obesity are insulin and the insulin receptor, glucose via GLUT1, and several stimuli activating TLR4 and 2. IR, insulin receptor; GLUT, glucose transporter; TLR, Toll-like receptor; mTOR, mammalian target of rapamycin; HIF1a, hypoxia-inducible factor 1a; HATs, histone acetyl transferases; TCA, tricarboxylic acid; a-KG, alpha ketoglutarate; KDM, lysine demethylase; EHMT2, Euchromatic Histone Lysine Methyltransferase 2.

Several of the metabolic pathways mentioned above are also critical to regulate trained immunity in monocytes and macrophages. Upon the first encounter with a training stimulus, there is a profound rewiring of various intracellular metabolic pathways, including glycolysis, OXPHOS, glutaminolysis, cholesterol synthesis, and fatty acid metabolism.^26^ These pathways are important for the energy production that is necessary for innate immune memory, but they also regulate biosynthesis, proliferation, and impact on intracellular signalling pathways. For example, up-regulation of these pathways is essential for the induction of epigenetic changes via supply of specific metabolites or by regulating epigenetic enzymes licensing subsequent increased response upon secondary stimulation^5^ (*Figure 2*).

Glycolysis is one of the two canonical pathways for ATP synthesis in immune cells. During glycolysis, glucose is converted into pyruvate, which then either enters the TCA cycle in the mitochondria for further processing or is converted to lactate. Although glycolysis is classically the primary source of energy in anaerobic conditions, it is also highly up-regulated in activated immune cells^25^ to offer a faster (but less efficient) supply of energy and additional metabolic products. Up-regulation of glycolysis is also essential for the induction of trained immunity via activation of the Akt–mTOR–HIF1a pathway.^9^ After stimulation by various training stimuli, including β-glucan,^9^ BCG,^27^ and endogenous stimuli such as oxLDL^28^ and catecholamines,^29^ aerobic glycolysis is up-regulated evidenced by up-regulated glycolytic enzymes,^9^ increased uptake of 13C-radiolabeled glucose,^27^ as well as increased glycolytic activity measured with Seahorse technology.^28,29^

In addition to rapid energy production, up-regulation of glycolysis also promotes the conversion of NAD+ to NADH, which is an important co-factor for many enzymes. Furthermore, glucose-6-phosphate can fuel the PPP for the production of nucleotides and NADPH. In addition, whereas lactate was long seen as by-product of glycolysis, lactate can directly bind to histones and modify the epigenetic landscape of macrophages stimulated with LPS, at least in mice.^30^ Inhibition of glycolysis, either directly by 2-deoxy glucose or by 3-(3-pyridinyl)-1-(4-pyridinyl)-2-propen-1-one (3-PO), which inhibits the inducible PFK-2/FBPase isozyme 6-phosphofructo-2-kinase/fructose-2,6-biphosphatase 3 (PFKFB3), abolishes the up-regulated cytokine production, the hallmark of trained immunity.^28^ Another line of evidence establishing the relevance of this pathway comes from the presence of single nucleotide polymorphisms in key glycolytic genes in healthy human subjects affecting the induction of trained immunity *ex vivo*.^27,28^

In addition to glycolysis, trained macrophages are characterized by increased oxygen consumption, indicative of higher OXPHOS. Macrophages trained by β-glucan, BCG, oxLDL, and catecholamines show an augmented basal and maximum oxygen consumption rate 6 days after initial exposure.^27–29,31^ Pharmacological inhibition of OXPHOS, e.g. by the complex V inhibitor oligomycin, prevents trained immunity by oxLDL^32^ and β-glucan,^31^ but not by BCG.^27^ Specifically for β-glucan, it has been shown that this increased OXPHOS is the consequence of epigenetic activation of specific enzymes, including succinate dehydrogenase B, by up-regulation of the methyltransferase Set7.^31^

Trained immunity is also critically dependent on glutamine replenishment of the TCA cycle.^8^ In monocytes stimulated with β-glucan or BCG, glutaminolysis is up-regulated, which subsequently leads to an accumulation of fumarate, succinate, and malate. Inhibition of the conversion of glutamine into glutamate can prevent trained immunity. Fumarate itself can induce training in isolated human primary monocytes, whereas malate and succinate are unable to promote training. Furthermore, fumarate inhibits KDM5, a histone 3 lysine demethylase, promoting the persistence of histone trimethylation at the promotor regions of pro-inflammatory genes (see Section 2.3).

Centrally located at the cross-roads of various metabolic pathways that are important for inducing trained immunity is acetyl-CoA. First, in the cytosol, acetyl-CoA feeds into the cholesterol synthesis pathway via the conversion to mevalonate. β-Glucan-trained cells showed an up-regulation of the cholesterol synthesis pathway and we have shown that trained immunity critically depends on the activation of this pathway.^7^ Inhibiting the conversion of mevalonate by HMG-CoA reductase inhibitors prevents trained immunity. Mevalonate itself can induce training via activation of the Insulin-like Growth Factor 1 Receptor and subsequent mTOR activation. This leads to the enrichment of the activating histone modification histone 3, lysine 4 trimethylation (H3K4me3) on pro-inflammatory genes, ultimately leading to enhanced pro-inflammatory cytokine production upon restimulation.^7^ The same pathway is also essential for reprogramming haematopoietic stem cells into a trained phenotype.^33^

In addition, acetyl-CoA can be used for FAS.^25^ β-Glucan trained macrophages show an up-regulation of the FAS pathway, but pharmacological inhibition of this pathway during the 24 h exposure to β-glucan did not interfere with trained immunity.^8^ For training with the endogenous adrenal hormone aldosterone, however, up-regulation of the FAS pathway via the mineralocorticoid receptor is critical for the increased production of IL-6 and TNF-α.^34^

### 2.3 Epigenetic remodelling in trained immunity

In addition to adaptations to intracellular metabolism, trained immunity is also regulated via epigenetic rewiring. The molecular mechanisms of this rewiring are only partially understood, but there is evidence for several layers of regulation, including histone modifications and subsequent changes in the accessibility of chromatin, as well as transcription of long non-coding RNAs.^35^ In addition, DNA methylation is increasingly recognized for a role in the establishment of innate immune memory.^21^

In resting immune cells, pro-inflammatory genes are repressed or ‘closed’ via repressive histone marks and DNA methylation. Following immune cell activation, repressive marks are rapidly removed, and activating histone marks such as methylation of histone 3 lysine 4 (H3K4me3) and acetylation of H3K27 are added, making the DNA accessible for transcription factors. After the binding of transcription factors, chromatin loops are formed, allowing for tighter regulation of gene transcription via topologically associated domains (TADs). Finally, microRNAs regulate degradation of the RNA after transcription, adding another layer of regulation.

During the establishment of innate immune memory, several histone modifications are important for enhanced activation upon restimulation. After initial stimulation of the immune cell, the epigenetic landscape does not return to baseline (i.e. before stimulation), but it leaves an ‘epigenetic scar’. For example, upon β-glucan stimulation, increased levels of the activating histone modification H3K4 monomethylation (H3K4me1) are observed on distal enhancers, H3K4me3 on active promotor sites, and H3K27ac on both active enhancers and promotors.^36^ Co-incubation of monocytes with inhibitors of methylation and acetylation during training prevents the induction of trained immunity.^9,36^ In addition, decreased H3K9me2, which is a repressive mark normally closing the chromatin for transcription, is also implicated in the regulation of trained immunity at least for BCG training.^35,36^ Due to the combination of these ‘epigenetic scars’, gene transcription is rapidly enhanced upon secondary stimulation, leaving the cells more responsive.

Chromatin modifications are regulated via histone-modulating enzymes, such as methyltransferases or demethylases. Three enzymes have been identified that are critical in the induction of trained immunity: the lysine demethylase KDM5, which is inhibited by the up-regulation of fumarate in the TCA cycle,^8^ the lysine methyltransferase (KMT) Set7, which methylates H3K4me1 at the enhancer regions and is up-regulated in trained immunity by β-glucan,^31^ and lysine methyltransferase G9a/EHMT2, which methylates H3K9me2 leading to gene inaccessibility and is down-regulated upon trained immunity by BCG.^37^ In addition, other histone modifications could be important in the induction of trained immunity, such as histone lactylation or methylation/acetylation on other lysine residues. More research is needed to further unravel epigenetic mechanisms of trained immunity and their regulation for therapeutic potential.

Not only histone modifications, but also DNA methylation has increasingly been recognized to modulate trained immunity. In *in vitro* studies using β-glucan to induce training or LPS to induce tolerance, DNA methylation patterns did not change upon β-glucan stimulation, but they did upon LPS tolerance, suggesting a larger role for DNA methylation in suppression of gene expression rather than in increase.^36^ However, in monocyte-derived macrophages from BCG-vaccinated healthy human adults, alterations in DNA methylation were associated with enhanced anti-mycobacterial immunity *ex vivo*.^38^ And in addition, very recently, DNA methylation was described as the underlying driver for long-term (>1 year) non-specific beneficial effects of BCG vaccination in neonates.^21^ DNA methylation was established approximately 6 days after initial exposure to BCG *in vitro* and was preceded by H3K27ac, indicating a connection between DNA and histone modifications. Further research into the stimulus-specific DNA methylation changes and the interaction with histone modifications is, therefore, warranted.

On an even deeper molecular level, long non-coding RNAs (lncRNAs) and TADs are also important in trained immunity regulation. Their role in trained immunity is discussed in recent excellent literature.^5^ In short, some regions of the DNA have enriched chromosomal contacts through the folding of the chromatin, leading to so-called TADs. LncRNAs can modulate the contact through the formation of chromosomal loops, bringing functionally related genes into proximity, subsequently leading to gene transcription.^39^ LncRNAs are now emerging as novel modulators of the long-term epigenetic reprogramming of innate immune cells and are called immune priming lncRNAs (IPLs). To date, the role of IPLs and lncRNAs is only described for training by β-glucan, but it might very well be an important epigenetic mechanism also for other inducers of trained immunity.

## 3. The role of inflammation and innate immune cells in atherosclerotic CVD, obesity, and diabetes (*Figure 3*)

### 3.1 Systemic inflammation in atherosclerosis

The past decade has witnessed a gradual change in the contribution of specific ‘traditional’ risk factors in driving atherosclerotic cardiovascular disease (ASCVD).^40^ In many countries, the number of people who smoke has decreased. In addition, due to statins, ezetimibe, and proprotein convertase subtilisin-kexin type 9 (PCSK9) inhibitors, circulating concentrations of low-density lipoprotein (LDL)-cholesterol are drastically reduced in patients at high cardiovascular risk, which lowers the contribution of LDL-cholesterol to ASCVD progression. In contrast, other risk factors gained significance. Due to their strongly increasing prevalence, obesity, metabolic syndrome, and type 2 diabetes mellitus rapidly gain importance as drivers of ASCVD. The metabolic syndrome (defined according to the National Cholesterol Education Program ATP III criteria as the presence of at least three of the following five traits: abdominal obesity, increased serum triglycerides, low HDL-c, increased blood pressure, and hyperglycaemia) serves as an indicator of obesity-related metabolic dysregulation and is strongly associated with ASCVD.^41^ Also, there is now strong evidence that triglyceride-rich lipoproteins, which are often increased in patients with metabolic syndrome and diabetes, are causally related to ASCVD development.^42^ Interestingly, these and other changes in risk factors are associated with time-dependent changes over the period of one decade in the histological composition of carotid plaques, which were removed by endarterectomy.^43^

**Figure 3:**
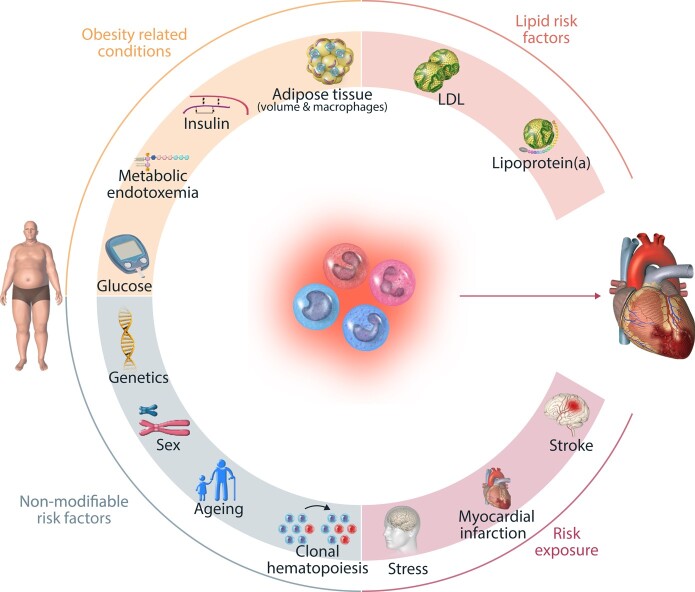
Factors leading to inflammation in cardiometabolic disease. Many risk factors for cardiometabolic disease can influence inflammation and subsequently CVD. Obesogenic risk factors include adipose tissue volume and macrophages, insulin levels, metabolic endotoxaemia, and glucose levels. In addition, several lipid risk factors, LDL, and Lipoprotein(a) are associated with inflammation. Other risk exposures are stress and myocardial infarction and stroke itself, and finally, the non-modifiable risk factors are genetics, sex, age, and related clonal haematopoiesis.

In addition to this shift in traditional risk factors contributing to ASCVD, inflammation and immune activation have been identified as important atherogenic mechanisms. Inflammation is one of the mechanisms linking various traditional risk factors to ASCVD, but it can also drive atherosclerosis development independent of these risk factors, e.g. in the context of clonal haematopoiesis,^44^ and as a mechanism that increases cardiovascular risk in patients with chronic inflammatory disease such as rheumatoid arthritis or HIV. The causal role of inflammation in ASCVD has recently been established by randomized controlled trials with anti-inflammatory drugs. The ‘canakinumab anti-inflammatory thrombosis outcomes study’ (CANTOS) reported that the anti-interleukin 1β monoclonal antibody canakinumab lowered the risk for recurrent myocardial infarction, stroke, or cardiac death by 15% in patients with a previous myocardial infarction and a high-sensitivity C-reactive protein level of 2 mg or more per litre.^45^ More recently, two randomized controlled trials showed that also the non-specific anti-inflammatory drug colchicine, traditionally used for the treatment of gout attacks, reduced the major adverse cardiovascular event rate in patients with coronary artery disease.^46,47^

### 3.2 Innate immune cell activation in atherosclerosis, obesity, and diabetes

**Figure 4:**
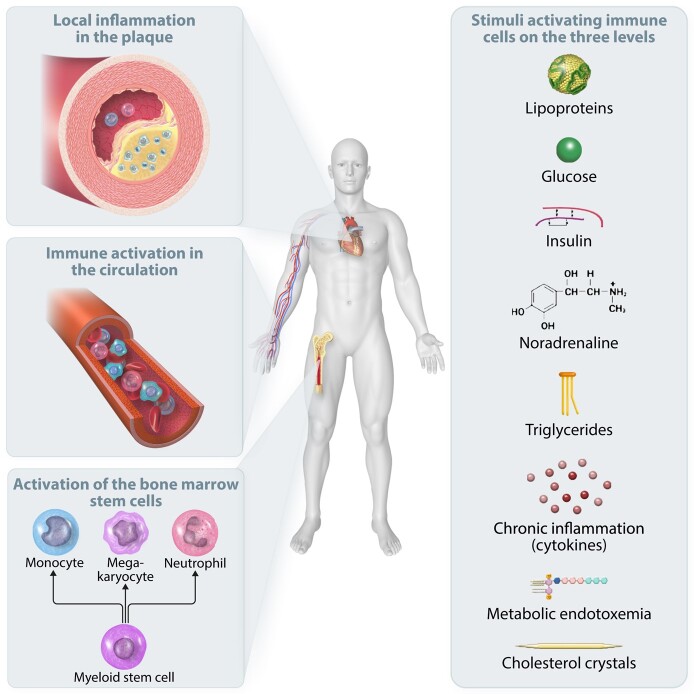
Three levels of inflammatory activation in ASCVD. Inflammation in patients with ASCVD can occur on three levels: in the bone marrow, where progenitor cells are stimulated and skewed towards the myeloid lineage; in the circulation, where circulating metabolites activate circulating immune cells; or in the plaque, where local damage-associated molecular patters and environmental cues such as hypoxia can activate and reprogram infiltrating innate immune cells or local tissue-resident macrophages.

Innate immune cells importantly contribute to the systemic inflammation observed in the context of ASCVD. Monocyte-derived macrophages are the most abundant immune cells in atherosclerotic plaques and orchestrate the initiation, progression, and destabilization of these plaques. Strategies that prevent the accumulation of monocytes into plaques strongly limit atherosclerotic plaque formation in animal studies.^48^ More recently, the contribution of circulating neutrophils to the atherosclerotic process has been recognized, which is discussed in detail in other reviews.^49^ There is a strong correlation between circulating numbers of monocytes, neutrophils, and the neutrophil-to-lymphocyte ratio and the occurrence of ASCVD.^50^ After entering the intima, monocytes differentiate into macrophages that contribute to the formation of the atherosclerotic plaque by engulfment of modified lipoproteins (such as oxidized LDL particles) and subsequent foam cell formation; in addition, macrophages produce chemokines and cytokines after stimulation by several damage-associated molecular patterns that are present in the plaque micro-environment and are sensed by macrophage pattern recognition receptors, including Toll-like receptors (TLRs).^51^ Importantly, in patients with established coronary artery disease, also the circulating monocytes, before entering the atherosclerotic plaques, are characterized by a pro-inflammatory phenotype, producing more inflammatory chemokines and cytokines after *ex vivo* stimulation.^52,53^ The progenitors of these circulating monocytes in the bone marrow [the haematopoietic stem and progenitor cells (HSPCs)] have the same hyperresponsive phenotype in patients with coronary artery disease and show myeloid skewing.^54^ Similar findings were obtained in patients with increased ASCVD risk due to hypercholesterolaemia: their circulating monocytes produce more cytokines after *ex vivo* stimulation and the HSPCs show a hyperinflammatory phenotype.^55^ These findings illustrate that in patients with ASCVD or with traditional risk factors predisposing to ASCVD, activation of the innate immune system not only occurs in the micro-environment of the atherosclerotic plaque, but also in the circulation and bone marrow niche (*Figure 4*).

The presence of obesity has a profound impact on our immune system. In addition to changes in absolute cell numbers, phenotypes of immune cells are also affected leading to a state of chronic low-grade inflammation.^56^ There is a significant correlation between obesity and diabetes and circulating leucocyte numbers, particularly of the myeloid lineage, and weight loss decreases monocyte and neutrophil numbers.^57^ The circulating leucocyte number predicts the incidence of type 2 diabetes in a population-based cohort.^58^ In children with obesity, the number of circulating monocytes is increased and obesity is associated with increased CD14^++^ monocyte numbers and an activated phenotype of the CD14^++^ monocyte subsets.^59^ This chronic pro-inflammatory state is involved in driving various obesity-associated complications including the development of insulin resistance and atherosclerosis.^60^

One of the key drivers of this chronic low-grade inflammation is the expanding adipose tissue, which is accompanied by a profound change in immune cell numbers and phenotypes. For example, the number of adipose tissue macrophages is known to increase robustly, and this is paralleled by a shift towards a more pro-inflammatory phenotype. These so-called metabolically activated macrophages residing in obese adipose tissue are characterized by increased expression of lipid-related genes and various pro-inflammatory markers.^61^ More downstream, these inflammatory markers drive various complications associated with obesity. For example, pro-inflammatory cytokines are known to interfere with the insulin signalling route ultimately leading to insulin resistance and type 2 diabetes.^62^

The presence of obesity leads to changes in various metabolic parameters including increased plasma glucose and insulin concentrations, changes in lipid concentrations, and alterations in adipokines secreted from obese adipose tissue. In addition, obesity can be accompanied by metabolic endotoxaemia.^63^ Several lines of evidence have suggested that these metabolic alterations are leading to long-term changes in a wide variety of cell types including innate immune cells and their progenitors.^64,65^ Moreover, these immunomodulatory changes appear to persist even after the obesity-associated metabolic alterations have been normalized. This suggests the presence of long-term immunological effects of metabolic dysregulation that can be explained by trained immunity driving long-term changes in innate immune cell function.^66,67^ Dysregulation of immune cell function ultimately leads to a chronic inflammatory state underlying the development of various complications associated with obesity.

In the next paragraphs, we will discuss in detail how factors associated with ASCVD, and in particular, obesity- and diabetes-associated factors, can have long-lasting effects on leucocytes and bone marrow progenitors by trained immunity.

## 4. Trained immunity in ASCVD

### 4.1 Trained immunity by (modified) lipoproteins

LDLs are abundantly present in atherosclerotic plaques. Brief exposure of isolated primary human monocytes to low concentrations of oxLDL, and to a lesser extent also to acetylated LDL (acLDL), induces a trained immune phenotype.^68^ These oxLDL-trained macrophages are characterized by enhanced production of pro-inflammatory cytokines such as IL-6, MCP-1, and TNF-α, and increased expression of matrix metalloproteinases, after restimulation with TLR2 and TLR4 ligands. Furthermore, these trained macrophages are more amenable to foam cell formation via up-regulation of CD36, and scavenger receptor A, and a down-regulation of the cholesterol efflux transporters ABCA1 and ABCG1. Similar to β-glucan and BCG-induced-training, oxLDL-induced trained immunity is critically dependent on the up-regulation of glycolysis and OXPHOS and the enrichment of the activating histone modification H3K4me3 in the pro-inflammatory cytokine gene regions.^28,32,68^

Lipoprotein(a) is a lipoprotein consisting of an LDL-like lipoprotein coupled to an apolipoprotein(a) moiety that carries oxidized phospholipids. Elevated Lp(a) levels are independently associated with ASCVD such as coronary artery disease.^69^ In the same experimental design as used for oxLDL, 24 h exposure to Lp(a) dose-dependently induced trained immunity in isolated human monocytes, characterized by enhanced production of TNF-α and IL-6 after restimulation with LPS, which is mediated by the oxidized phospholipids carried by the Lp(a).^70^

The *in vitro* findings on the training capacity of oxLDL and Lp(a) have successfully been translated to patients with elevated circulating concentrations of LDL-c and Lp(a). Isolated monocytes from patients with treatment-naïve familial hypercholesterolaemia with severely elevated LDL-c concentrations showed a pro-inflammatory phenotype compared with matched normocholesterolaemic control subjects, with increased cytokine production capacity, transcriptomic changes in metabolic pathways, and the enrichment of the histone marker H3K4me3. Despite the fact that *in vitro* oxLDL-induced trained immunity can be prevented by statins, a 3-month course of lipid-lowering therapy with statins did not reverse this trained immune phenotype in patients with familial hypercholesterolaemia.^71^ In a more recent study, bone marrow aspiration was performed in patients with familial hypercholesterolaemia before the start of cholesterol-lowering therapy and 3 months after the start of the therapy. Bone marrow HSPCs from patients with familial hypercholesterolaemia showed increased gene expression in pathways involved in HSPC migration and myelomonocytic skewing. Three months of cholesterol-lowering treatment reverted the myelomonocytic skewing, but there was no change in the transcriptomic enrichment of monocyte-associated inflammatory and migratory pathways, indicating a memory of the previous high LDL-c.^55^

With regard to Lp(a), monocytes isolated from patients with elevated Lp(a) levels are pro-inflammatory, migrate faster through endothelial cell layers, and after radioactive labelling and auto-transfusion, demonstrate an increased homing to the arterial wall compared with controls with low levels of Lp(a).^70^

The significance of trained immunity in ASCVD is also highlighted by its presence in patients with established ASCVD. The first indications for a trained immune phenotype in the context of atherosclerosis in humans *in vivo*, were observed in 2016 in a small cross-sectional study in patients with symptomatic coronary artery disease compared with healthy controls.^52^ Monocytes from these patients showed up-regulated cytokine production capacity, accompanied by an increased expression of glycolytic enzymes, as well as the typical enrichment of H3K4me3 on the promotors of cytokine genes indicative of trained immunity. A more recent study in a similar patient population confirmed the circulating monocyte reprogramming and also demonstrated in CD34+ HSPCs, isolated by a bone marrow aspiration, marked functional and transcriptomic reprogramming towards the myeloid lineage.^54^

### 4.2 Diet-induced trained immunity

A few experimental studies have reported that specific diets [e.g. Western-type diet (WD)] or specific diet-related metabolites can induce trained immunity. In LDL-receptor knockout mice (*Ldlr -/-*), a 4 week WD induces hypercholesterolaemia, systemic inflammation, and activation of the innate immune system.^72^ Four subsequent weeks of chow diet following the WD completely normalizes the systemic inflammation, but leaves the myeloid cells in a persistent hyperresponsive phenotype upon restimulation with TLR ligands. As an explanation for the long-lasting presence of these hyperresponsive monocytes in the circulation, their bone marrow progenitor cells, particularly granulocyte-monocyte progenitors (GMPs), were shown to be reprogrammed on a transcriptional and epigenetic level. Mechanistically, the NLRP3 inflammasome and subsequent production of IL-1β are essential in inducing this memory phenotype.^72^ Although in this particular study, atherosclerosis was not assessed as an outcome, previous studies have shown that transplantation of bone marrow from WD-fed *Ldlr-/-* mice into chow-fed recipients increases atherosclerotic lesion size independent of serum cholesterol levels.^73^ Similarly, Seijkens *et al*.^74^ showed that hypercholesterolaemia can induce priming of HSPCs in *Ldlr-/-* mice, skewing them into the myeloid lineage, which persists when transferred to normocholesterolaemic mice. Ultimately, transplantation of hypercholesterolemia-primed HSPCs leads to aggravation of atherosclerosis in normocholesterolaemic *Ldlr-/-* mice via up-regulated inflammation, indicative of a pro-inflammatory HSPC memory.

High-fat diets can increase gut permeability with translocation of LPS to the systemic circulation, which is called metabolic endotoxaemia. In *ApoE-/-* mice, intermittent administration of a very low amount of LPS, mimicking this metabolic endotoxaemia exacerbated atherosclerotic lesions and induced trained immunity.^75^ Upon short-term stimulation, monocytes polarized into a sustained pro-inflammatory state, as shown by increased Ly6C, CCR5, and MCP-1 expression, ultimately leading to aggravated atherosclerosis several weeks after the initial stimulus. To demonstrate the causal role of monocyte reprogramming, the authors performed adoptive transfer of *ex vivo* LPS-reprogrammed monocytes once weekly for 1 month, which also increased plaque size.^76^

A recent study in healthy Tanzanians identified other diet-derived circulating metabolites that can modulate trained immunity. The study compared urban with rural-living individuals in 232 healthy Tanzanians. Urban living was associated with an increased pro-inflammatory cytokine production capacity of isolated peripheral blood mononuclear cells (PBMCs).^77^ Several food-derived metabolites were identified that accounted for these differences by directly modulating the cytokine production capacity of circulating monocytes. Interestingly, serum from the urban-living individuals induced trained immunity when added to healthy donor monocytes *ex vivo*. This was prevented by apigenin, which is a dietary-derived flavone, with higher circulating concentrations in rural-living individuals since it is found in high concentrations in the traditional rural Tanzanian diet like in stable cereal millet and sorghum. Apigenin also tended to prevent oxLDL-induced trained immunity in isolated human monocytes *in vitro*.

### 4.3 Trained immunity induction by hyperglycaemia

One of the key metabolic complications associated with obesity-induced insulin resistance is elevated plasma glucose levels. High plasma glucose concentrations are a risk factor for the development of various complications including atherosclerosis.^78^ Both acute and long-term effects of glucose on innate immune cells have been identified that appear to persist after lowering plasma glucose levels. Epidemiological studies have initially coined the term ‘hyperglycaemic memory’ to describe the observation that a period of high blood glucose levels (hyperglycaemia) is somehow memorized in individuals with diabetes.^79^ Even after lowering blood glucose levels, this metabolic memory towards a previous hyperglycaemic episode translates into an increased risk for the development of ASCVD. More recent studies have shed light on potential underlying molecular pathways involving changes in innate immune cells as well as non-immune cells with immune function (such as endothelial cells) that may partly explain these observations.

Endothelial cells are one of the first sensors of harmful endogenous stimuli such as high glucose concentrations in the blood. They were also the first cells in which mechanisms of hyperglycaemic memory were discovered. Already in 2008, El-Osta *et al*. showed that endothelial cells are highly sensitive to glucose fluctuations, resulting in an enhanced inflammatory response due to epigenetic changes such as increased H3K4me1 on pro-inflammatory genes. When the endothelial cells were subsequently exposed to normoglycaemic conditions, the epigenetic changes and enhanced inflammatory response remained.^80^ Since then, many additional studies have unravelled pathways, epigenetic enzymes, and additional molecular mechanisms of endothelial cell memory.^81^

More recent studies focused on the effects of hyperglycaemia on myeloid progenitor cells in the bone marrow. In streptozotocin-induced diabetic mice, increased numbers of circulating neutrophils and Ly6-C(hi) monocytes were observed resulting from activation of myelopoiesis in the bone marrow.^82^ Follow-up studies showed that hyperglycaemia induces differences in macrophages originating from these progenitor cells. This became apparent by comparing bone marrow-derived macrophages isolated from normoglycaemic vs. streptozotocin-induced hyperglycaemic mice. After *ex vivo* differentiation of these bone marrow cells in normoglycaemic medium, macrophages originating from hyperglycaemic bone marrow were characterized by increased pro-inflammatory gene expression and proatherogenic characteristics.^64^ This was partly explained by changes in metabolic signatures of the cells illustrated by an increase in the glycolytic rate of the cells that is known to drive inflammatory traits of macrophages,^83^ and also licenses the development of trained immunity. Moreover, transplantation of bone marrow from diabetic mice into normoglycaemic *Ldlr*-/- recipient mice that were subsequently fed a WD aggravated atherosclerosis development, with also an increased proportion of plaque macrophage nuclei staining positive for H3K4me3. Further analysis revealed involvement of the Runt-related transcription factor 1 (Runx1) in translating hyperglycaemia into persistent proatherogenic changes of the bone marrow.^64^

In addition to chronic hyperglycaemia during diabetes, observational studies have shown that variations in plasma glucose levels also have harmful effects.^84–86^ Glucose variability including short-term increases in plasma glucose levels is prevalent in individuals with impaired glucose metabolism and has been shown to increase the risk for ASCVD. Mimicking this so-called *transient intermitted hyperglycaemia* (TIH) in *Apoe*-/- mice by weekly intraperitoneal injections of glucose accelerated atherogenesis. This was largely driven by an increase in myelopoiesis in the bone marrow, resulting in increased circulating monocytes and neutrophils. Deletion of S100a9 and S100a8, produced by activated neutrophils, prevented the increase in circulating monocytes and reduced TIH-induced atherosclerosis.^65^

Interestingly, TIH did not lead to increased levels of HbA1c that is used to monitor long-term plasma glucose levels. This suggests that additional readouts beyond HbA1c are needed to monitor plasma glucose levels and variability. The current widespread use of continuous glucose monitoring devices has opened up new lines of research to understand the impact of glucose variability on immune cell function and the development of CVD in humans with diabetes.

Besides the effects of glucose on myeloid progenitor cells, high glucose concentrations have also been shown to promote trained immunity in human mature monocytes. Exposure of isolated primary human monocytes to 25 mM of glucose for 24 h followed by 5 days of culturing in normoglycaemic conditions led to increased production of TNF-α after stimulation with LPS. These effects involved hyperglycaemia-mediated epigenetic changes through regulation of the mixed lineage leukaemia family.^66^ Epigenetic changes induced by high glucose levels may also be conveyed by additional pathways including the histone methyltransferases SMYD3 and SET7/9.^87^

While training and epigenetic effects may lead to long-term changes in innate immune cells, direct effects of high levels of glucose on immune cells are also well known. Exposure of macrophages to high glucose concentrations is known to drive more pro-inflammatory responses illustrated by enhanced release of various pro-inflammatory cytokines.^88,89^ This may in part be explained by an increase in the glycolytic rate of the cells after exposure to hyperglycaemic conditions leading to the pro-inflammatory response of macrophages.^64,90^

It is important to emphasize that additional more indirect pathways may exist to explain the harmful effects of hyperglycaemia and glucose variability. High glucose levels are known to modulate proteins through a non-enzymatic reaction leading to advanced glycation end-products (AGEs).^91^ These irreversible modifications impact on the functional properties of proteins. In addition, a specific receptor exists that binds these AGEs. This receptor for AGEs is also expressed on immune cells including macrophages and its activation promotes pro-inflammatory responses.

Altogether, short- or long-term elevated glucose concentrations may have both direct and persistent effects on our innate immune system that remain even after lowering plasma glucose levels.

Other diabetes-related factors may also promote changes in immune cell function. The development of insulin resistance initially leads to higher plasma insulin levels to overcome the resistance state. Although insulin is not needed for glucose uptake of immune cells, the insulin signalling cascade is present in monocytes and macrophages and can impact their functional output. Insulin treatment of immune cells activates the PI3K/Akt/mTOR pathway in monocytes and leads to changes in the metabolism of the cells. This is paralleled by functional changes including increased cytokine production and enhanced migration capacity of the monocytes.^92^ Insulin may also act together with other metabolites to increase the inflammatory trait of monocytes. Indeed, a combination of high levels of insulin and saturated fatty acids activates human monocytes to produce pro-inflammatory cytokines.^93^ In addition, insulin enhances LPS-stimulated transcription of TNF-α, interleukin-6, and IL-1β in macrophages.^94,95^ In contrast, potential anti-inflammatory effects of insulin have also been reported. Insulin negatively regulates NFκB-activation,^96^ a key transcription factor activating pro-inflammatory gene expression. In the presence of high concentrations of glucose, insulin treatment of macrophages reduces LPS-activated gene expression of pro-inflammatory cytokines and lowers NO synthesis.^97^ Also, insulin may impact on immune cell function through metabolic effects since it has been suggested to regulate macrophage lipid metabolism.^98^ Although these immunomodulating effects of insulin might also modulate trained immunity, detailed mechanistic studies are currently lacking and warranted.^99^

### 4.4 Obesity and trained immunity

In addition to the existence of a metabolic memory towards individual metabolic cues associated with obesity, including glucose, it also seems that a period of obesity itself is memorized and leads to persistent changes in immune cell phenotype and function.

Although losing body weight is effective to reduce obesity-associated metabolic complications, body weight loss is often followed by weight regain.^100^ Approximately 80% of individuals who achieve a weight loss of >10% will regain their body weight within 1 year.^101^ Complete or even partial weight regain leads to worsening of metabolic complications compared with the previous period of obesity.^102–104^

Recent mechanistic studies may have shed some light on potential underlying mechanisms to explain this phenomenon. On the level of the adipose tissue, weight loss has been shown to reduce the chronic inflammatory state of the adipose tissue.^105^ However, weight loss does not completely normalize the inflammatory state since several immune markers remain elevated after weight loss.^106,107^ This so-called ‘obesogenic memory’ is characterized by an increase in pro-inflammatory macrophages that fail to normalize in number and phenotype after weight loss. These persistent changes despite subsequent weight loss appear to set the stage for a worsened response upon weight regain. Recent work has shown that subsequent weight regain worsens the inflammatory response in adipose tissue compared with a previous obesity period.^108^ This obesity-induced imprinting of adipose tissue immune cells promoted activation of antigen-presenting cells and enhanced lipid handling in macrophages in animals undergoing body weight fluctuation. It is important to note that body weight fluctuations also impacted on adaptive immune cell function including T cell exhaustion. On a systemic level, these changes lead to worsening of metabolic complications compared with obese animals not undergoing body weight fluctuations. Effects of body weight cycling even appear to go beyond the adipose tissue itself. For example, persistent changes in the microbiome induced by body weight cycling have been shown to drive worsening of metabolic complications.^109^

It also seems likely that changes in bone marrow cells induced by body weight cycling may contribute to enhanced activation of innate immune cells and worsening of metabolic complications. As discussed earlier, it has already been shown that communication between the adipose tissue and bone marrow exists driving enhanced myelopoiesis during obesity.^57^ How these pathways are affected by body weight cycling is currently unknown; however, one might speculate that weight cycling would lead to persistent or even more robust increase in myelopoiesis worsening the pro-inflammatory state associated with obesity. More studies are needed to decipher the existence of a metabolic memory towards other metabolites or proteins associated with obesity.

### 4.5 Trained immunity and uric acid

Serum uric acid is the end-product of purine metabolisms in humans, and high uric acid concentrations predispose to gout. Epidemiological studies have shown strong associations between hyperuricaemia and obesity, diabetes, hypertension, and ASCVD. Importantly, *in vitro* exposure of isolated primary human monocytes to soluble uric acid for 24 h enhances the cytokine response to immediate subsequent exposure to LPS, which is called immune priming, and is also dependent on histone methylation.^110^ This is distinguished from trained immunity by the shorter persistence of the priming effect: when the uric acid-primed cells were stimulated with LPS 24 h after removal of the uric acid, the IL-1β production was no longer increased, although for IL-6 a higher production was still observed even after 5 days of rest between the uric acid removal and LPS restimulation.^111^ Isolated PBMCs from patients with gout showed enhanced *ex vivo* production of IL-1β, which was associated with serum uric acid levels.^110^

### 4.6 Trained immunity by adrenal hormones: catecholamines and aldosterone

Another major risk factor for CVD is hypertension. Two hypertensive syndromes that are associated with a particularly increased CVD risk are primary hyperaldosteronism (PHA) and pheochromocytoma. PHA and pheochromocytoma are caused by excessive (adrenal) overproduction of aldosterone and catecholamines, respectively. Given the fact that cardiovascular morbidity and mortality in these patients are higher than in patients with essential hypertension with comparable blood pressure levels,^112,113^ we hypothesized that these adrenal hormones themselves could impact on innate immune cell function and induce trained immunity.

Brief exposure of isolated human primary monocytes for 24 h to relevant concentrations of aldosterone *in vitro* indeed induced a trained immune macrophage phenotype. This effect was abolished when cells were co-incubated with spironolactone, a mineralocorticoid receptor blocker.^34^ Surprisingly, aldosterone-trained macrophages showed different intracellular metabolic changes compared with oxLDL- or microbe-induced trained immunity. Instead of up-regulated glycolysis and OXPHOS, which were not observed, aldosterone exposure induced an up-regulation of FAS, which was due to the enrichment of H3K4me3 on the promoters of essential enzymes in FAS *FASN* and *ELOVL6*. Although isolated monocytes from patients with PHA did not show higher cytokine production capacity after *ex vivo* stimulation, monocyte-derived macrophages of these patients did show enhanced oxLDL-induced TNF-α expression.^114^

In a similar experimental model of *in vitro* training, the catecholamines adrenaline and noradrenaline induced trained immunity in isolated human primary monocytes.^29^ Interestingly, although concomitant administration of these catecholamines with LPS attenuated cytokine production in a short term, this cytokine production was enhanced when the cells were restimulated with LPS 6 days following catecholamine exposure. This was accompanied by an up-regulation of glycolysis and OXPHOS and was mediated via activation of the β1 and β2-adrenergic-cAMP pathways, and the enrichment of H3K4me3 on the promotor regions of cytokines and chemokines.

In patients with pheochromocytoma, a rare neuroendocrine tumour that releases bursts of catecholamines into the circulation, isolated monocytes showed trained immunity, characterized by increased cytokine production capacity, and enrichment of H3K4me3 at the promoter sites of pro-inflammatory genes. This trained immune phenotype sustained even 1 month after the surgical removal of the tumour, indicating a memory of the previous high catecholamine levels.^29^

In summary, monocytes from patients with these particular adrenal causes of hypertension have a trained immunity phenotype compared with blood pressure-matched subjects, and this is most likely due to the direct effects of the adrenal hormones. However, we have never studied trained immunity in the context of primary hypertension compared with normotensive control subjects.

Recent work also showed that psychosocial stress can accelerate atherosclerosis via the up-regulation of stress hormones and subsequent reprogramming of innate immune cells.^115^ Barrett *et al*. studied monocytes from stressed mice (and humans) and found a characteristic inflammatory transcriptomic signature and hyperresponsiveness upon TLR ligand stimulation, indicative of a trained immune phenotype. Transcriptomic and epigenomic analyses showed that the mTOR pathway was involved and that the chromatin accessibility was altered. Finally, myocardial infarction^116^ and stroke^117^ itself, both major stress-inducing events, lead to long-term reprogramming of HSPCs in the bone marrow via sympathetic nervous system signalling, ultimately aggravating atherosclerosis. However, it has not been investigated whether this is due to the typical metabolic and epigenetic mechanisms of trained immunity. The role of myeloid progenitor cells in trained innate immunity will be further discussed in the parallel review by Mitroulis *et al*. in this special issue.^15^

## 5. Future perspectives and clinical implications

With intervention studies reporting a reduction in the cardiovascular event rate in high-risk populations (typically after myocardial infarction) by anti-inflammatory drugs, the causal role of inflammation in ASCVD has been well-established. The recent ‘2021 ESC Guidelines on cardiovascular disease prevention in clinical practice’ included the recommendation to consider the treatment with colchicine to lower cardiovascular risk in secondary prevention of CVD, particularly if other risk factors are insufficiently controlled or if recurrent CVD events occur under optimal therapy (Class IIb, Level A evidence).^118^ Preclinical evidence summarized in this review suggests that inflammation is particularly contributing to ASCVD in the context of obesity, metabolic syndrome, and type 2 diabetes. In particular, there is accumulating evidence for innate immune cell reprogramming in patients with these conditions. Obesity itself, probably via inflammatory mediators from its adipose tissue macrophages, and factors associated with obesity, such as diet (WD,^72^ urban vs. rural diet^77^), hyperglycaemia,^64^ hyperuricaemia,^111^ dyslipoproteinaemia,^68^ and low-grade endotoxaemia,^75^ can induce long-lasting hyperresponsiveness of the innate immune system, by reprogramming of their bone marrow progenitors, which facilitates atherosclerosis. Trained immunity, through metabolic and epigenetic reprogramming, appears to be one of the mechanisms that contribute to this long-lasting immune cell activation. Only recently, Edgar *et al*. provided solid proof that trained immunity (in this particular study induced by hyperglycaemia) can indeed accelerate atherosclerosis development in a mouse model.^64^ Although monocytes from patients with coronary artery disease or with risk factors for ASCVD have markers of trained immunity, the causal role of trained immunity for atherosclerosis and ASCVD (both for endogenous atherosclerotic stimuli and for trained immunity induced by infections) has yet to be proved.

In the future, these novel insights into the role of the innate immune system might have several clinical implications. First, markers of trained immunity, e.g. epigenetic markers in circulating monocytes, might be associated with cardiovascular risk in certain patient populations, which would allow improved risk stratification. A hint that certain histone modifications might be associated with the development of diabetic complications has been reported in a subgroup of patients who participated in the Epidemiology of Diabetes Interventions and Complications (EDIC) study.^119^ Monocytes from patients with type 2 diabetes with a history of high HbA1c levels who had developed microvascular complications showed a greater number of promoter regions with enrichment in the active histone mark H3K9Ac compared with patients with a history of low HbA1c and without any microvascular complications.

A second future implication is that the knowledge that trained immunity contributes to the cardiometabolic and cardiovascular complications of obesity might improve personalized pharmacological prevention and treatment strategies. On the one hand, this can improve the prediction of treatment efficacy of known anti-inflammatory drugs, and on the other hand, trained immunity offers novel pharmacological targets. A hint of how trained immunity can be used to predict the efficacy of immunomodulatory drugs can be derived from a *post hoc* analysis of the CANTOS trial. It was suggested that patients with clonal haematopoiesis due to TET2 driver mutations, which is associated in experimental models with enhanced inflammasome activation and IL-1β production, may respond better to treatment with canakinumab than patients without clonal haematopoiesis.^120^ One could imagine that also the presence of trained innate immune cells might be associated with a stronger beneficial effect of immunomodulator drugs, such as colchicine. Importantly, IL-1β is also an important mediator of trained immunity. *In vitro* concomitant administration of IL-1 receptor antagonists prevents oxLDL-induced trained immunity and *in vivo* WD-induced trained immunity can be prevented by inhibition of the NLRP3 inflammasome.^72^

In addition to predicting the potential effects of existing drugs, the metabolic and epigenetic mechanisms driving trained immunity provide novel pharmacological targets. Specific compounds that inhibit the inducible glycolytic enzyme PFKFB3 can lower glycolytic rate without completely blocking glycolysis,^121^ and this prevents trained immunity *in vitro*.^28^ Inhibitors of glutaminolysis have been developed in the cancer field, and these drugs might also prevent trained immunity. In addition to metabolic modulators, trained immunity could also be suppressed by counteracting specific epigenetic changes with compounds such as histone methylation inhibitors. For an overview of epigenetic drug discovery, we refer to previous extensive reviews on this topic.^122,123^ The molecular and epigenetic intracellular processes driving trained immunity also have important physiological functions in immune cells and all other cells of the human body. Therefore, to provide specificity and limit adverse events, it is important to specifically target the myeloid cells that contribute to CVD. For this purpose, nanoparticles have been developed, for example, ApoA-I-based, HDL-like nanoparticles, that specifically deliver the chemical compounds they are loaded with, to myeloid cells.^124^  *In vitro* HDL-nanobiologics loaded with the mTOR inhibitor rapamycin down-regulate trained immunity induction in human isolated monocytes. In a mouse transplantation model, trained immunity induction occurs in graft-infiltration macrophages, and targeting this with rapamycin-loaded HDL-nanobiologics significantly improves graft survival, illustrating the potential of such therapies.^125^

Another way to interfere with trained immunity while avoiding overwhelming immunosuppression would be to specifically target those cells that show the most vigorous trained immunity phenotype. An important recent discovery is that not all monocytes respond similarly to a training stimulus, but instead three subgroups could be identified: (i) macrophages with enhanced expression of genes encoding chemokines and pro-inflammatory cytokines; (ii) macrophages with enhanced expression of chemokines only; and (iii) non-trained cells, with an approximate equal distribution.^126^ This finding suggests that specifically interfering with trained immunity in a subgroup of monocytes (comparable to specifically removing senescent immune cells with senolytics) is a future possibility.

There are some exciting and pressing open questions about trained immunity in the context of obesity and its metabolic and cardiovascular complications. First, it is important to unravel in more detail how trained immunity contributes to CVD in the setting of obesity and diabetes and to identify which subset of monocytes is trained, and how these cells can be identified. Secondly, we should explore the interrelatedness between trained immunity and other recently identified mechanisms that contribute to innate immune cell activation, such as clonal haematopoiesis, and immunosenescence. Furthermore, there is recent evidence in mice that trained immunity, at least induced by micro-organisms, is transmitted across generations.^127^ This might lead to an exciting new hypothesis to explain the link between maternal obesity and cardiovascular health in their offspring.^128^ Finally, to fully exploit the therapeutic possibilities related to trained immunity, it is important to also investigate trained immunity development in other cell types than monocytes. There is accumulating evidence that trained immunity can occur in NK-cells, innate lymphoid cells, and neutrophils.^129^ In addition, innate memory characteristics are observed in epithelial cells and endothelial cells, and it would be of great interest to further explore this in the setting of obesity, diabetes, and CVD.
